# Maggie’s Big Toe

**Published:** 1888-12

**Authors:** 


### MAGGIE'S BIG TOE.

Our readers are doubtless familiar with what have come to be known as the Rochester knockings. They first occurred some forty years ago at Hydesville, an outlying hamlet of Rochester, N. Y., and completely knocked out some very knowing ones with quite original theories. Among other investigators there was a committee of eminent Buffalo surgeons and physicians, who *interviewed* the two little Foxes who were thought to be in some manner responsible for the disturbances. This committee reported that the raps were made by the two bones of the leg being brought into sudden contrast. A good many people believed this, and then again, a good many didn't. The dids and the didn'ts have never quite agreed, but now it turns out after all this lapse of time, that nobody was exactly right. The Foxes, after the manner of their kind, were too cunning, and the Buffalo committee were too aspiring ; they sought a solution quite above the real facts ; in other words, they very naturally looked too high. Maggie has explained the whole business to a select audience of her friends : It was simply and singly her *big toe!* When the later committee of physicians examined her a few days ago, upon drawing off her stocking and holding their ears to the nail, they could hear a muffled sound, like the frictional sawing of two fragments of dry parchment, but the audience, even those at a distance in the wings and galleries of the great play house devoted to this Sunday evening performance, heard, at various nearby points, distinct raps, the exact counterpart of the old time Rochester knockings. There can be but a single explanation of this : Maggie's great toe is a *ventriloquist*, but ventriloquism is common at our dime museums, and the exposition of great toes merely, in these days of the undress drama and ballet festivities, fail to draw an admiring audience after a first performance. So Maggie has withdrawn her big toe, for the present, from the dramatic field, but we are not able to state but that it is still in training for other ventriloqual feats, should our flippant Richmond take the field anew.

Nothing could be more convincing, *absolutely nothing.*

---

Chicken Cutlets.—Chop the white meat of a four-pound (boiled) chicken, rather fine. Put a half pint of cream on to heat in a double boiler. Rub together one large tablespoonfulf of butter and two *rounding* ones of flour. When smooth, stir it into the cream, and stir and cook till it forms a thick paste ; add the unbeaten yolks of two eggs ; cook one minute longer. Take from the fire ; add the chicken, twelve mushrooms and six truffles, chopped fine, a quarter of a nutmeg, grated, ten drops of onion juice, a tablespoonful of chopped parsley, a dash of cayenne and a palatable seasoning of salt. Mix all well together and turn out to cool. When cold, form into cutlets, about the size and shape of a French chop ; dip first in egg and then in bread crumbs, and fry in smoking hot fat. Serve with cream sauce.

